# Reduced Overhead Routing in Short-Range Low-Power and Lossy Wireless Networks †

**DOI:** 10.3390/s19051240

**Published:** 2019-03-12

**Authors:** Muhammad Omer Farooq, Dirk Pesch

**Affiliations:** 1 Nimbus Research Centre, Cork Institute of Technology, Cork T12P928, Ireland; 2 School of Computer Science and IT, University College Cork, Cork T12P928, Ireland; d.pesch@cs.ucc.ie

**Keywords:** low-power and lossy networks (LLNs), short-range wireless networks, routing protocol, probabilistic forwarding

## Abstract

In this paper we present enhanced routing protocol for low-lower and lossy networks (ERPL), a reduced overhead routing protocol for short-range low-power and lossy wireless networks, based on RPL. ERPL enhances peer-to-peer (P2P) route construction and data packet forwarding in RPL’s storing and non-storing modes of operation (MoPs). In order to minimize source routing overhead, it encodes routing paths in Bloom Filters (BF). The salient features of ERPL include the following: (i) optimized P2P routing and data forwarding; (ii) no additional control messages; and (iii) minimized source routing overhead. We extensively evaluated ERPL against RPL using emulation, simulation, and physical test-bed based experiments. Our results demonstrate that ERPL outperforms standard RPL in P2P communication and its optimized P2P route construction and data forwarding algorithms also positively impact the protocol’s performance in multi-point to point (MP2P) and point to multi-point (P2MP) communications. Our results demonstrate that the BF-based approach towards compressed source routing information is feasible for the kinds of networks considered in this paper. The BF-based approach results in 65% lower source routing control overhead compared to RPL. Our results also provide new insights into the performance of MP2P, P2MP, and P2P communications relative to RPL’s destination-oriented directed a-cyclic graph (DODAG) depth, i.e., a deeper DODAG negatively impacts the performance of MP2P and P2MP communications, however it positively impacts P2P communication, while the reverse holds true for a relatively shallow DODAG.

## 1. Introduction

Typically, Wireless Sensor Networks (WSNs), Internet of Things (IoT), and Cyber-Physical Systems (CPS) all require low-power and lossy networks (LLNs) for some or all of their operation. These networks are characterized as LLNs because nodes in such networks possess limited resources and often operate in harsh communication environments, resulting in transmission losses over wireless links. Communication and computing devices are increasingly being embedded in objects and structures to enable networked sensing and actuation functions. This is resulting in sophisticated CPS, such as power generation and distribution networks, assisted living, traffic control, home automation, safety systems, autonomous vehicles, and distributed robotics. Unlike the pure sensing focus of traditional WSNs, CPS require distributed decision making, control, and actuation. Hence, low power devices need to communicate not only with a central gateway, but also need to directly communicate with other sensor or actuator devices. Here, we refer to these types of communication as multi-point to point (MP2P), point to multi-point (P2MP), and peer-to-peer (P2P) communications.

Due to energy and radio communication limitations, devices in a LLN may not connect directly to the gateway or to all other devices in the same network, but collaborate to relay or multi-hop data packets wirelessly to each other and to the gateway. Hence, a routing protocol is required to discover MP2P, P2P, and P2MP data forwarding paths. Considering the limitations of LLNs, the Internet Engineering Task Force (IETF) ROLL (routing over low-power and lossy networks) working group has standardized the routing protocol for low-lower and lossy networks (RPL) [1]. RPL supports MP2P, P2MP, and P2P route discovery and data forwarding. Moreover, it is a generic routing framework for LLNs and uses the concept of an objective function (OF) to influence how routes are constructed in a particular LLN. If there is no RPL OF defined in the standard that meets the requirements of a particular LLN and/or to satisfy the requirements of application(s), a new OF can be designed. Hence, the concept of OFs adds flexibility, scalability, and adaptability to RPL. Due to these reasons, RPL has emerged as a standard and widely adopted routing protocol for IPv6/6LoWPAN-based networks. Considering the popularity of RPL, recent research has also focused on using RPL in low-power wide-area networks, an emerging networking technology for IoT [2]. Existing RPL design handles many of the challenges imposed by LLNs, however in this paper we further enhance RPL to satisfy the requirements of emerging CPS and IoT use cases.

Despite RPL’s flexibility, here we highlight some of its inefficiencies, such as sub-optimal P2P route discovery and data forwarding, and use of source routing for P2MP and P2P communications in RPL’s non-storing mode of operation (MoP). We present enhancements to improve P2P route construction and data forwarding in RPL’s storing and non-storing MoPs, and we also present a bloom filter (BF) based routing and data packet forwarding approach to minimize source routing overhead. We capture these enhancements in an improved protocol, called Enhanced RPL (ERPL). The following are our main contributions:

(1) Identification of inefficiencies in RPL’s route construction and data forwarding algorithms.
(2) Improvements to P2P route construction and data forwarding algorithms for RPL’s storing and non-storing MoPs.
(3) A Bloom Filter based approach for P2MP data forwarding to minimize source routing overhead in RPL’s non-storing MoP.
(4) Analyzing the impact of RPL’s destination-oriented directed a-cyclic graph (DODAG) depth on MP2P, P2MP, and P2P communications.
(5) An analysis of the protocol using emulation, simulation, and test-bed based experiments. Our results show that ERPL demonstrates overall lower packet loss, delay, requires fewer transmissions to deliver P2P packets, lower control overhead, and is more energy-efficient compared to RPL.

The remainder of this paper is organized as follows: RPL’s brief introduction is presented in Section 2. Related work is discussed in Section 3, and our reduced overhead routing protocol for low-power short-range lossy wireless networks is presented in Section 4. Performance evaluations are presented in Section 5, and finally conclusions are presented in Section 6.

## 2. RPL: Routing Protocol for Low-Power and Lossy Networks

RPL [1] is an IETF standardised routing framework for LLNs. Generally, in a RPL network there is a border router also known as a gateway/root that connects different nodes in the network to an external network, such as the Internet. Within a network, RPL supports MP2P, P2MP, and P2P communications. RPL constructs a routing topology called destination-oriented directed a-cyclic graph (DODAG) rooted at the gateway. To discover data forwarding paths, RPL uses the following control messages:

(1) DIO: DODAG Information Object (used to discover forwarding paths for MP2P communication)
(2) DIS: Destination Information Solicitation (a node multicasts this message to discover the route to the gateway)
(3) DAO: Destination Advertisement Object (used to discover forwarding paths for M2MP and P2P communications)

### 2.1. DODAG Construction

Initially, the gateway/root multicasts the DIO message. Upon receipt of the DIO message, direct neighbors of the gateway decide to join the DODAG advertised in the DIO message based on their Objective Function (OF). If a node joins the DODAG, using the RPL trickle timer [1], the node also multicasts the DIO message. This process continues and eventually DODAG information reaches all the nodes in a network. If any node in the network is not interested in joining the DODAG advertised in the DIO message, the node ignores the message. Multiple DODAGs can exist in the same network, and they are differentiated by their instance ID.

### 2.2. P2MP and P2P Route Construction and Data Packet Forwarding

RPL can work in two MoPs, namely: (i) storing mode, and (ii) non-storing mode. As the name implies, in the non-storing MoP, nodes do not store and maintain routing table for P2P communication. If a node has a data packet for another node, it forwards the packet to the gateway/root, and the gateway uses source routing to forward the packet to the destination node. RPL uses DAO control messages to construct P2P and P2MP forwarding paths. In the non-storing MoP, nodes use MP2P forwarding to send their DAO packet to the root. In this way, the root discovers forwarding paths to different nodes in a network. In the storing MoP, each node maintains data forwarding paths to the other nodes in its sub-DODAG, and this is achieved through the transmission of DAO packets. Whenever a node needs to forward a data packet to another node in its network, the node uses the information in its forwarding table to relay the packet to the destination node. If the route to the destination is not present in the forwarding table, the packet is transmitted to the node’s parent node. The process continues until the common ancestor is reached, and from there the packet is relayed to the destination node.

## 3. Related Work

In [3], the authors argue that interoperability between RPL’s storing and non-string MoPs can result in a network that is more effective and fault-resilient. First, the authors demonstrate that coexistence of RPL’s storing and non-storing MoPs in the same network can lead to different problems, such as network partitioning and routing loops. Then different enhancements to the RPL protocol are proposed. The modified protocol resolves the identified problems and facilitates interoperability between RPL’s MoPs in the same network. Another approach to the coexistence of RPL’s storing and non-storing MoPs is presented in [4]. The drawback of the stated approaches is that the MoP is configured statically, hence nodes cannot switch between MoPs dynamically. To overcome memory limitations of RPL’s storing mode, a modification to the RPL protocol called D-RPL is proposed in [5]. D-RPL overcomes the limitation by replacing storing mode forwarding with multicast dissemination. If a node’s memory limits are reached, D-RPL activates to overcome the limitation.

In [6], an analysis of the RPL protocol from an Internet of Things (IoT) perspective is presented. The analysis considers the following benchmarks: reliability and robustness, mobility, resource heterogeneity, and scalability. The article concludes that to remain a viable option in the IoT domain, RPL needs improvement, especially in P2P communications, to support emerging IoT use cases. Experimental performance evaluation of RPL using hop-count and/or ETX routing metrics is presented in [7,8,9], and RPL performance evaluation for multi-gateway networks using different objective functions is presented in [10]. To reactively discover P2P communication paths, enhancements for RPL are proposed in [11,12]. However, the enhancements require additional control messages and substantial changes to the RPL routing protocol.

In [13], a mechanism is presented to create a software-defined WSN. In a software-defined WSN, a gateway/controller needs to have complete information about a network’s topology. To enable the controller to discover the network topology, each node in the network transmits addresses of its direct neighbors to the controller. A node in a typical WSN can have tens of neighbors, therefore each node transmitting IPv6 or IEEE 802.15.4 MAC address of each of its direct neighbors results in considerable overhead in such a network. To eliminate the stated problem, each node in a network uses a Bloom Filter (BF) based approach to transmit information about its direct neighbors to the controller. The protocol presented in the paper is only limited to topology discovery at the controller.

In [14], an opportunistic routing protocol based on RPL is presented. Instead of using unicast, the protocol proposes the use of anycast for MP2P forwarding. In this case, whenever a node receives an anycast message, and if the rank of the node is less than the rank of the transmitting node, the node forwards the packet and sends an ACK to the transmitting node. Instead of using RPL’s unicast DAO message for P2P route discovery, the protocol proposes the use of a routing set. A node periodically broadcasts its routing set using RPL’s trickle timer, and the routing set contains information about the nodes in the node’s sub-DODAG. If the network topology is known at the time of the network deployment, the routing sets are constructed using a bitmap. However, if the network topology is not known at the time of network deployment, and if the topology can change dynamically, the protocol compresses the routing set information in a BF. Therefore, nodes in a network do not need to store a traditional routing table, instead they store routing sets. This results in lower memory requirements to forward P2P data packets. However, this technique can only work in RPL’s storing MoP. In the non-storing MoP, for P2MP and P2P data forwarding the protocol still uses source routing.

In [15], a behavioral study of RPL under network partitioning is presented. This work demonstrates that TinyRPL and ContikiRPL implementation do not function as expected under network partitioning. Based on RPL’s standard specification, the authors model protocol’s dynamic behavior, and it has been demonstrated that a standard compliant implementation of RPL functions as expected under network partitioning.

In [16], an end-to-end route registration for P2MP communication and a novel policy to manage neighbor tables in RPL are presented with an aim to enhance RPL’s scalability in dense and large networks. It is shown through experiments that the new features improve RPL’s packet reception rate in large and dense networks.

The lack of mechanisms towards emerging security threads in LLNs pose a significant challenge in the continued adoption of RPL. To mitigate security challenges, a number of enhancements to RPL are proposed. In [17], an algorithm is presented to identify misbehaving nodes in a RPL network. Each node in a network monitors its preferred parent forwarding behavior to observe the packet loss rate. Each node compares the observed packet loss rate with the packet loss rate obtained from one-hop neighbors. Using this process, a node is bale to identify a misbehaving preferred parent node. In SPLIT [18] a secure extension to RPL is proposed. SPLIT uses a lightweight remote attestation technique to ensure software integrity of network nodes. SPLIT reduces the control overhead of remote attestation by piggybacking the attestation process onto RPL control messages. In [2], RPL is used to enable multi-hop communications in Long Range (LoRa); a wide-area low-power wireless networking technology. A new RPL OF is presented that aims to discover forwarding paths with minimum air time. Minimizing air time can result in lower energy consumption, a key feature in LLNs. It has been shown that enabling multi-hop communication in LoRa using RPL can support more IoT use cases compared to what can be supported with LoRaWAN’s traditional star topology.

The existing work on RPL largely focuses on protocol analysis for different scenarios, interoperability between storing and non-storing MoP, reducing memory requirement for the storing MoP, security enhancements, and reactive discovery of P2P data forwarding paths. Existing work on reactive discovery of P2P paths requires additional control overhead, which requires changes to the standard protocol and typically requires more memory, which can be problematic for resource-constrained nodes. Moreover, an approach that reduces the source routing overhead in RPL’s non-storing MoP does not exist. Therefore, this paper focuses on the following: (i) improved P2P routing and forwarding in RPL without using additional control messages and with minimal change to the protocol so as to maintain interoperability with standard RPL implementations, and (ii) reduced source routing overhead for P2MP and P2P communications in RPL’s non-sorting MoP. These aspects are largely missing in the existing literature. Furthermore, this paper extends the work presented in [19].

## 4. Reduced Overhead Routing Protocol

In this section, we present our enhanced routing protocol for short-range low-power and lossy wireless networks (ERPL). ERPL has the following two components:

(1) Enhanced P2P routing and forwarding
(2) Reduced overhead P2MP routing

### 4.1. Enhanced P2P Routing and Forwarding

#### 4.1.1. P2P Routing Problem in RPL

**Figure 1:**
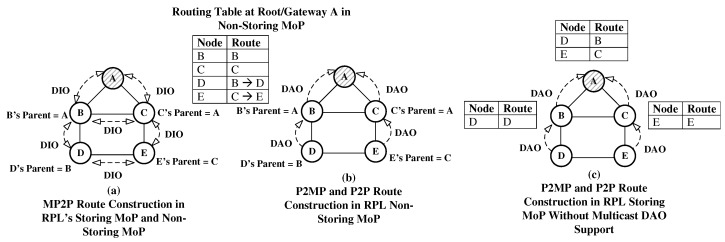
Routing Protocol for Low-Lower and Lossy Networks (RPL) Operation with Storing and Non-Storing Modes of Operation (MoPs).

RPL functionality in its different MoPs is shown in Figure 1. Here, our assumption is that the RPL Objective Function (OF) 0 [20] is in use. OF 0 uses shortest hop-count as the routing metric. RPL DODAG construction by exchanging DIO control messages is shown in Figure 1a. Using RPL’s OF 0, nodes in the network select their preferred parent, and consequently MP2P routes are constructed.

The route construction process followed by RPL’s non-storing MoP for P2MP and P2P data forwarding is shown in Figure 1b. During the route construction process, each node in a network transmits a DAO message to its parent node. Upon receipt of the DAO message, the parent node appends its own information to the message, and transmits the message to its parent. Finally, the DODAG’s root receives the DAO message. Using the DAO message, the root stores and maintains a forwarding path for each node in a network. If any node needs to communicate with another node in a network, the node’s data packet is transmitted to the root, and using the source routing the root forwards the packet to the destination node. We further elaborate the process with the help of the following scenario: node D needs to transmit data packet to node E; as nodes do not store a forwarding table in the non-storing MoP, node D forwards the data packet to the root (node A), and the root uses source routing to deliver the packet to E. Hence, in this case, the packet needs four transmissions (D⇒B⇒A⇒C⇒E) regardless of the fact that node D and node E are direct neighbors. Furthermore, there is an additional control overhead as source routing requires additional bits to be transmitted by each node along the path from the root to the destination node. It is well known that wireless transmission and reception consume most of a node’s energy. Hence, in this scenario RPL consumes more energy than needed as it not only requires extra transmissions, but also incurs extra control overhead due to source routing.

P2MP and P2P route construction in RPL’s storing MoP is shown in Figure 1c. It should be noted that Figure 1c demonstrates the functionality of the existing RPL implementations in storing MoP. In storing MoP, each node advertises routes to the nodes in its sub-DODAG, for example, a DAO message by node D for its parent node (B) contains information about its reachability. Afterwards, node B stores a forwarding table entry for node D, B transmits the same information in a DAO message to its parent node (A). In this process, B and A learn a data forwarding path to D. Using the same process, all nodes in a network transmit DAO messages to the root. For P2P forwarding, a node consults its forwarding table, and if the path to the destination node is known, it forwards the packet to the next hop, otherwise the data packet is forwarded to the node’s parent. This process continues until a common ancestor or root forwards the packet to the destination node. In our example, if node D needs to communicate with node E, the packet again needs four transmissions (D⇒B⇒A⇒C⇒E) regardless of the fact that D and E are direct neighbors. Therefore, data packet forwarding in RPL’s storing MoP is also not efficient. In this case, it is also feasible that node E needs to communicate with Node B. Using the stated algorithm, the data packet travels on the following path (E⇒C⇒A⇒B), despite node B being a direct neighbor of node C. Ideally, node C should directly transmit the packet to node B instead of forwarding it to node A. This can lower the number of transmissions to deliver the packet, hence lower energy consumption and contention in the network.

**Figure 2:**
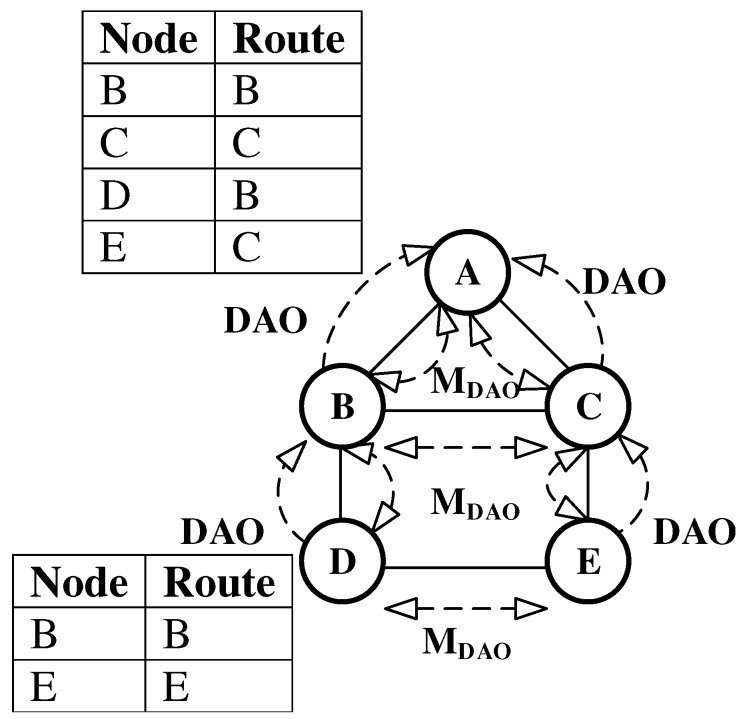
Point to Multi-Point (P2MP) and Peer-to-Peer (P2P) Routing Using Multicast Destination Advertisement Object (DAO).

The RPL standard proposes the use of multicast DAO $\left(M_{DAO}\right)$ messages to eliminate the deficiencies presented above. Nodes multicast $\left(M_{DAO}\right)$ messages so that nodes in the network can maintain forwarding table entries for their direct neighbors. The P2MP and P2P route construction process using $M_{DAO}$ messages is shown in Figure 2. The figure only shows forwarding tables at root A and node D. Now, if node D needs to communicate with node E, it can directly forward the packet to node E resulting in lower number of transmissions. As this approach uses an extra control message apart from DIO and unicast DAO messages, it is also not as energy efficient as it could be.

#### 4.1.2. Proposed Enhancement

To enhance RPL’s P2P route construction and data packet forwarding algorithms, we present an enhancement to current algorithms with the objective to avoid extra transmissions when a destination node is a direct neighbor of a source node or the destination node is a direct neighbor of a node relaying a data packet. Our enhancement can satisfy the stated objective by avoiding RPL’s $M_{DAO}$ message. Furthermore, no change is needed to RPL’s control messages. Due to the stated features, our proposed solution can easily coexist with existing RPL implementations.

The DIO control message is used by RPL to construct MP2P routes. Corresponding to different DODAGs, DIO messages are multicast in the network. If a node becomes part of a DODAG advertised in a DIO message, the node also multicasts the DIO message corresponding to that DODAG using RPL’s trickle timer. If a node does not become part of a DODAG, it ignores the DIO message. The above explanation shows that in any case nodes in a network have to process the DIO message. Regardless of whether a node joins a DODAG or not, each node receives DIO messages mulitcasted by its direct neighbors due to the broadcast nature of the wireless channel. A node can use its neighbor’s DIO message to discover its direct neighbors and it can also store forwarding table entries for them in its forwarding table. Thus, instead of using a $M_{DAO}$ message to discover and maintain forwarding table entries for direct neighbors, the same can be achieved by using DIO messages and implementing the following modifications:

(1) If a DIO message is received at a node, the node extracts the multicasting node’s address from the DIO message, and stores it in its forwarding table. This step is carried out without considering whether the node is interested in joining the DODAG or not. If an entry for the multicasting node is already present in the table then the entry is refreshed and the next hop field is set equal to the multicasting node’s address. If a node has a packet to transmit, it searches the destination address in its forwarding table. If the corresponding entry is available in the forwarding table, the packet is transmitted to the destination node or to the next hop. Otherwise, RPL forwarding rules are followed.
(2) The above algorithm works in storing MoP, but nodes do not maintain forwarding tables in the non-storing MoP. In non-storing MoP, whenever a node receives a DIO message, it searches the IPv6 neighbor table. If the DIO message’s multicasting node is not present in the table, a new record for the multicasting node is created and stored in the neighbour table. In this case, a NULL value is stored in the neighbor table’s field for the link-local address. This forces the MAC layer to obtain a link-local address for the neighbor node before transmitting a frame. In non-storing MoP, another approach can be used that can store the multicasting node’s address at the networking layer. In the non-storing MoP, this is feasible as nodes only need to store their direct neigbours’ network layer address. In a realistic scenario nodes typically have resources to store addresses of their direct neighbors. If a node requires to transmit a packet, the node searches the destination node’s address in its neighbor table or the data structure holding addresses of the node’s direct neighbors. If a match exists, the packet is forwarded to the destination node, otherwise the RPL’s forwarding algorithm for non-storing MoP is followed. When the root forwards the packet, it uses source routing, hence the presented forwarding algorithm only works if source routing is not used, i.e., when a P2P communication packet has not reached the root.

**Figure 3:**
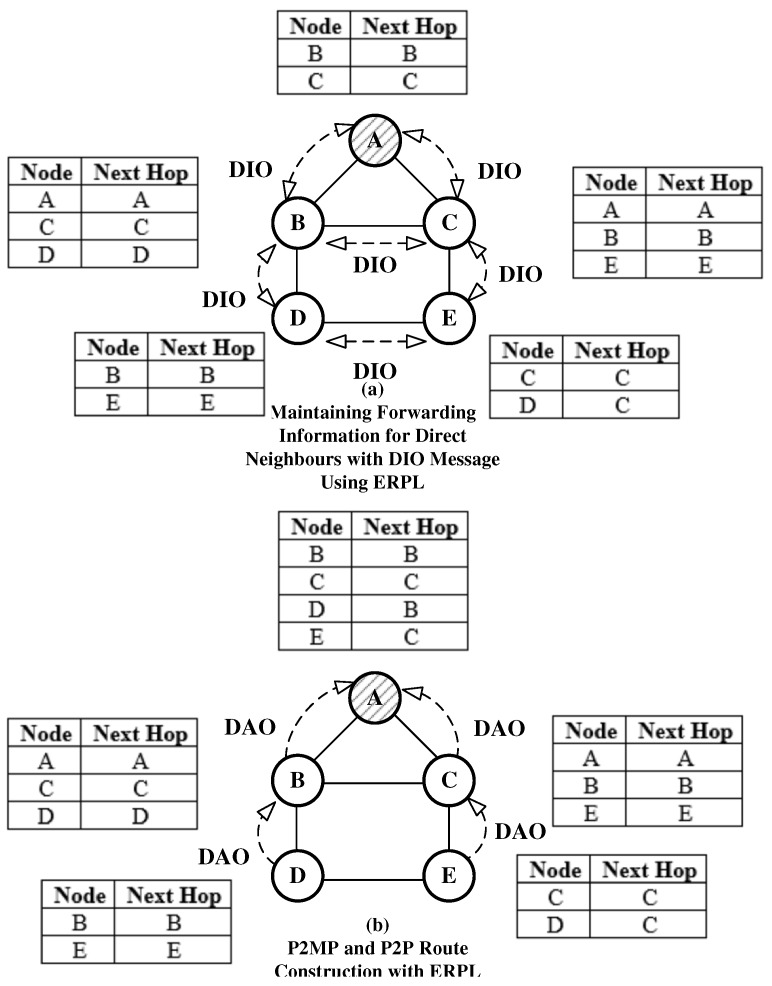
P2MP and P2P Route Construction in Enhanced RPL (ERPL).

ERPL use of DIO message to maintain direct neighbors’ information is shown Figure 3a. Similarly, the process of P2MP and P2P route construction is shown in Figure 3b. The figures demonstrate that ERPL can achieve the same functionality with reduced control overhead. A comparison of Figure 3 with Figure 1 reveals that ERPL can achieve the same functionality with fewer transmissions and lower control overhead.

**Figure 4:**
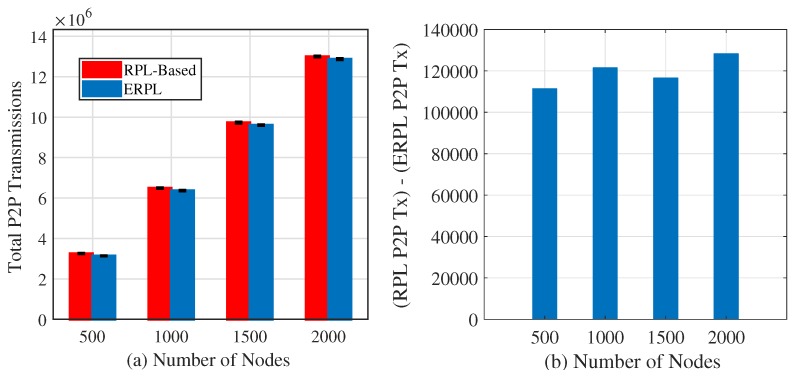
P2P Transmissions Comparison.

To develop a better understanding of ERPL’s potential benefits, we carried out computer-based simulation experiments. In experiments, the number of nodes in the network is varied from 500 to 2000. In our simulated network topologies, any node can have at most 8 direct neighbors, and the maximum number of hops between a node in a network and the root node is no more than 6 hops. In general however, a node can have an arbitrary number of neighbors using ERPL, and similarly a node can have an arbitrary number of nodes along the forwarding path to the gateway. In our simulation, each node generates 1000 P2P packets, and the destination peer node for each packet is selected randomly. Each simulation was repeated 30 times. P2P transmission comparison for ERPL and RPL is shown in Figure 4a. The figure reveals that ERPL demonstrates statistically significantly lower number of P2P transmissions to deliver P2P data packets compared to RPL. The difference between both protocols’ P2P transmissions is shown in Figure 4b. The figure shows that in all cases RPL requires over 100,000 more transmissions compared to ERPL. Hence, ERPL can help to reduce a node’s energy consumption. Moreover, ERPL requires a lower number of transmissions to deliver P2P data packets, hence it has the potential to positively impact the performance of MP2P and P2MP communications due to lower contention in a network.

### 4.2. Point to Multi-Point Reduced Overhead Routing

In the non-storing MoP, RPL uses source routing for P2MP communication. Source routing results in variable size IP packet header, the variable size header can result in higher packet processing delay. Moreover, as IP addresses or IEEE 802.15.4 MAC addresses of all the nodes along the path to the destination are present in the packet header, this approach also results in a higher control overhead. Typically, nodes in a low-power wireless network possess limited energy, and it is well known that wireless transmission consumes most of a node’s energy. Therefore, source routing in such networks results in higher power consumption, hence shorter network lifetime and frequent replacement of nodes’ energy sources.

To circumvent the issues associated with RPL’s source routing in P2MP communication, we present a forwarding protocol that relies on a probabilistic data structure called a Bloom Filter (BF). BF is a time and space efficient m-bit array data structure. A BF uses *k* hash functions to insert an element into the BF. The *k* hash functions are individually applied to an element that needs to be inserted in the BF, and each hash function individually sets a bit in the m-bit array. To check whether an element is in the BF, the same *k* hash functions are applied to the element to obtain *k* positions in the m-bit array. If the corresponding *k* bits in the m-bit array are set, the element may exist in the BF, however if any of the *k* bits is not set, the element is certainly not in the BF. However, BFs can yield false positive results. A BF configuration includes the set {k,m,n}. We have already discussed the set’s elements *k* and *m* but have not yet discussed the element *n*. *n* represents the maximum number of items that can be inserted in the BF’s m-bit array. The probability of false positive depends on the values of the elements in the set {k,m,n}. Equation (1) shows the probability of false positives for specific values of *k*, *m*, and *n*. An effective and efficient network design for a short-range low-power and lossy wireless network use case, such as smart city, smart home, surveillance, etc., invariably does not result in a network in which every node is many hops away from the root. Therefore, in this scenario, if we replace source routing information with BF’s m-bit array data structure, the value of *n* is certainly not large for the type of network considered here. In this research, we use n=12 as it is a big enough value for the type of networks we are targeting here, and the same value is also suggested in [21]. For a false positive probability of approximately 1%, the following two configurations yield values of *k* equal to 4 and 2 respectively: (i) n=12 and m=128 bits and (ii) n=12 and m=256 bits.

(1) $$p={\left(1-{\left(1-\frac{1}{m}\right)}^{kn}\right)}^{k}$$

#### 4.2.1. Choice of the Hash Functions

Typically, nodes in a short-range low-power and lossy wireless network possess limited resources, such as memory, and processing capabilities. Therefore, for the research domain considered here, we need hash function(s) with low memory complexity. To limit the packet forwarding delay the hash functions(s) should be simple enough so that hashes can be computed quickly in resource constraint nodes. Moreover, to be used in a BF such a hash function should also possess the following properties: (i) uniformity, (ii) applicability, (iii) versatility, and (iv) efficiency. We choose the Shift-Add-XOR (SAX) hash function class due to its simplicity and it also possesses the above mentioned properties [22]. Moreover, it works with any type of data with a similar efficiency. A BF uses *k* hash functions each producing a hash value of $log_{2}\left(m\right)$ bits. To simplify this, a single hash function can be used to produce $k\times log_{2}\left(m\right)$ bits, and afterwards the output can be split into *k* equal partitions, as suggested in [14].

**Figure 5:**
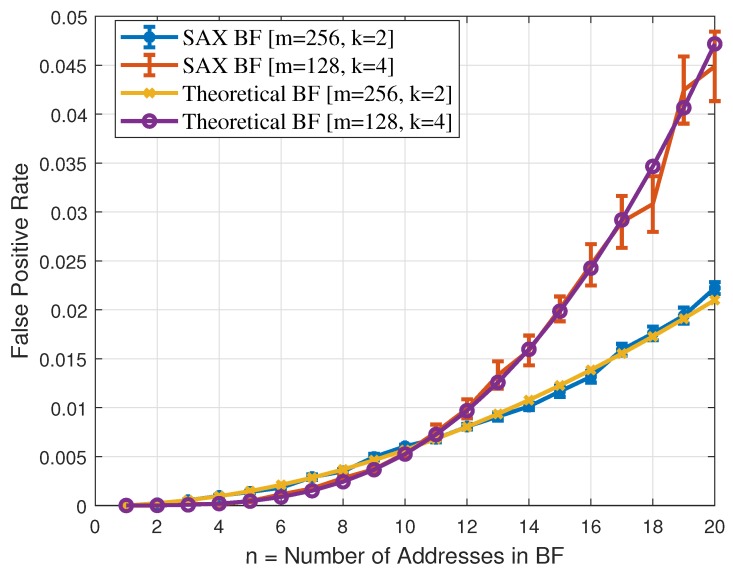
Bloom Filter False Positive Rate Comparison.

To analyze the feasibility of using BF for compressing the source routing information in a packet header, we need to determine a BF’s false positive rate using the SAX hash function. Hence, we carried out a number of experiments. In our experiments, we analyzed the following two BF configurations: (i)m=128bits,k=4 and (ii)m=256bits,k=2. We varied *n* from 1 to 20 and used IEEE 802.15.4 MAC addresses. To determine the BFs’ false positive rate, we generated 1000 random IEEE 802.15.4 MAC address in each experiment and applied SAX hash function to obtain *k* positions in the BF’s m-bit array. The values at those *k* positions then determine whether any of the addresses was present in the BF. We repeated each experiment 30 times and in order to obtain confidence intervals (CI) around the mean we used the t-distribution with the sample size of 30. Figure 5 shows the comparison of SAX false positive rate (using the two configurations discussed above) with the theoretical bound. Figure 5 reveals that there is a very close fit between SAX false positive rate and the theoretical bound, hence SAX hash function based bloom filter is a good choice for compressing the source routing information in the packet header.

#### 4.2.2. Data Packet Forwarding

Whenever the root node has a data packet to transmit to another node in the network, it uses the network topology information to construct the source routing path to the destination node. For each node along the path to the destination node (excluding the source node), the root passes the node’s address through the respective SAX hash function, and sets the resultant *k* bits in the bloom filter. Once the above process is complete for each node along the path to the destination node, the root inserts the resultant m-bit bloom filter array into the IP packet header, and forwards the packet to the next hop. When a node along the path to the destination receives the packet, it compares its address with the destination node’s address present in the IP header. If both addresses match, the packet is forwarded to the transport layer. Otherwise, the node consults its neighbor table, and retrieves neighbor node addresses from it one after another. If a retrieved neighbor node address is of the node from whom the node received the packet, the neighbor is ignored. This check is essential otherwise routing loops can be easily created in the BF-based P2MP routing. In IP data packet forwarding, a node does not have direct access to the forwarding node’s IP address, however as the forwarding node is a neighbor of the node who received the packet, the node can obtain the forwarding node addresses from its neighbor table. Moreover, to successfully complete the above check, the MAC layer passes the forwarding node network layer address to the networking layer. For all other neighbor addresses, the node uses the same SAX hash function to obtain *k* bit positions. Afterwards, the node checks whether the values at the obtained *k* positions inside the m-bit array present in the packet header are set. If bits at those *k* positions are set, the node forwards the packet to the neighbor after decrementing the hop limit field in the protocol header. However, if any of the bits at those *k* locations is not set, the node extracts the next neighbor node address from its neighbor table, and repeats the process. If after completing this process, the node does not find the next hop for the packet, the packet is dropped.

#### 4.2.3. Techniques to Reduce the Impact of False Positive on Routing

We are using a BF configuration that targets a maximum false positive rate of 1%. In order to reduce the impact of false positives on our P2MP routing approach, we also present different forwarding rules here.

**Rule-1:** If the destination node is the direct neighbor of the node that is processing the IP packet header, the node forwards the packet directly to the destination node without examining whether any of its neighbors are present in the BF.

**Rule-2:** If the source node is the direct neighbor of the node that is processing the IP packet header, the node ignores the source node’s address, i.e., it does not apply SAX hash function to the source node address.

**Rule-3:** Following the above rules may reduce the false positive rate, however routing loops can still occur due to the false positive characteristics of the BF. To mitigate the impact of forwarding a packet in a loop for long, we suggest the use of the IPv6 hop limit field. With the assumption that the root can reach any node in a network in at most *h* hops, the hop limit field can be set to the value of *h*. In the evaluation presented here we have assumed a value of h=12.

## 5. Performance Evaluation

In this section, we evaluate our ERPL protocol. First, we evaluate ERPL’s P2P routing and data forwarding protocols, and in the second sub-section, we evaluate ERPL’s P2MP routing and data packet forwarding protocols.

### 5.1. ERPL’s P2P Routing and Data Forwarding Performance Evaluation

Here, we present a simulation and physical test-bed based protocol performance evaluation. We implemented ERPL’s P2P routing and data forwarding algorithms in the Contiki operating system. Simulation-based performance evaluations were carried out using the widely used Cooja emulator [23] using the real program code for embedded devices. The test-bed based performance evaluation was performed using the FlockLAB [24] test-bed. The protocols use the objective function 0 [20]. In our Cooja setup, 50 Tmote sky motes were placed in a network that spans an area of 250 × 250 m${}^{2}$. There is a single gateway in our network. We evaluate the protocol’s performance using different random and grid topologies. Moreover, we analyze the impact of the gateway’s position and the nodes’ data generation interval on the protocols performance. The general simulation parameters are stated in Table 1. The total duration of a single simulation is 1020 s. We categorize our performance benchmarks as follows: reliability, latency, and energy consumption. For reliability we measure and report mean packet delivery ratio (PDR), for latency we report mean per-packet end-to-end delay, and for energy consumption we report the total number of times P2P communication packets are relayed and the total number of re-transmissions. We compare our ERPL protocol against a RPL protocol implementation. The experiments were performed using storing MoP, because in storing MoP nodes can forward packets to the destination largely without involving the root node, hence we can obtain an upper bound on the protocols’ performances.

For MP2P communication, each node generates data packets. Nodes start generating data packets at 10 simulation seconds, and stop the generation of data packets at 1000 s. For P2MP communication, the gateway starts generating data packets at 20 s, and stops the generation of P2MP data packets at 1000 s. For each data packet, the gateway selects a destination node at random. Similarly, for P2P communication, each node starts P2P communication at 20 s, and stops P2P packet transmission at 1000 s. For each data packet, nodes randomly select the peer destination node. We repeated our experiments by varying the MP2P, P2MP, and P2P communications packet generation interval. The packet generation interval is varied from 2 s to 8 s.

#### 5.1.1. Random Topology Results

We evaluate the protocols using 10 different fully connected random topologies. Moreover, to evaluate the gateway’s position impact on the protocols’ performances, two different sets of experiments were carried out. For the first set of experiments, the gateway is located at the edge of a network and for the second set of experiments the gateway is located at the center of a network.

**Figure 6:**
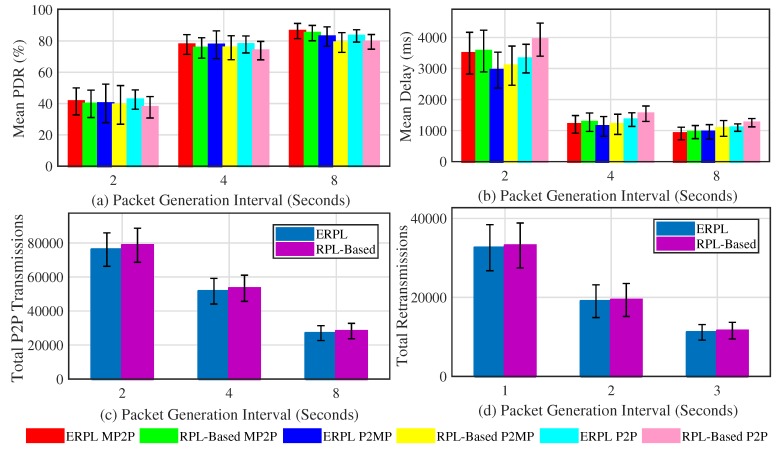
Protocols’ Performances with Different Random Topologies (Gateway at Network Edge).

Figure 6 demonstrates the protocols’ performances in random topologies when the gateway is placed at the edge of the network. The mean PDR for the protocols along with 95% confidence interval (CI) around the mean is shown in Figure 6a. In all sets of experiments, our ERPL protocol demonstrates higher mean PDR compared to the standard RPL-based protocol. For all considered communication types, ERPL demonstrated higher PDR, however the CIs overlap. The protocols’ performances change from one topology to another, hence the CIs overlap despite of the fact that ERPL demonstrated higher PDR in each random topology. Generally, as the packet generation interval increases the protocols achieved better PDR due to lower data contention. One of the objectives of ERPL is to focus on improving P2P routing and data forwarding, therefore it outperforms standard RPL by a higher margin in P2P data forwarding compared to the other two types of communication. Figure 6a also shows that the improved P2P routing and data forwarding mechanisms of ERPL has a positive impact on MP2P and P2MP communications.

The mean per-packet delay demonstrated by the protocols in P2MP, MP2P, and P2P communications is shown Figure 6b. Generally, in comparison to RPL, ERPL demonstrated lower mean per-packet end-to-end delay for different communication types. For MP2P, P2P, and P2MP communications, the CIs corresponding to the protocols overlap despite the fact that ERPL demonstrated lower delay in the different random topologies. This is due to the reason that both protocols’ performances differ from topology to topology, hence the CIs overlap. Our improved P2P data packet forwarding algorithm also results in lowering the per-packet end-to-end delay for MP2P and P2MP communications.

The protocols’ comparison with regard to the number number of times P2P packets are forwarded in reaching their destination is shown in Figure 6c. Similarly, Figure 6d shows the re-transmission comparison. Using ERPL the number of times P2P packets are relayed in the network is lower compared to the RPL protocol. Furthermore, the number of re-transmissions by ERPL is also lower. As ERPL relays P2P packets less, it demonstrates better PDR, delay, and total number of re-transmissions.

**Figure 7:**
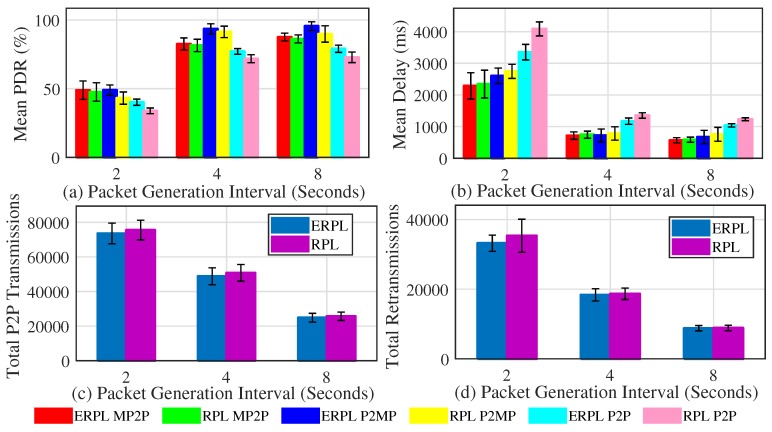
Protocols’ Performances with Different Random Topologies (Gateway at Network Center).

The protocols’ performances for different random topologies with the gateway placed at the center of the network is shown in Figure 7. The mean PDR achieved by both protocols corresponding to different types of communication is shown in Figure 7a. Generally, trends present in Figure 7a are similar to the trends present in Figure 6a, i.e., ERPL achieves better PDR in different types of communication, and it significantly outperforms RPL in P2P communication. The comparison of Figure 6a and Figure 7a sheds light on an interesting insight, i.e., when the gateway is placed at the center of the network, the PDR achieved by the protocols corresponding to MP2P and P2MP communications is slightly higher and the PDR demonstrated by the protocols corresponding to P2P communication is slightly lower compared to the scenario when the gateway was at the edge of the networks. Average MP2P and P2MP route length is usually shorter in case the gateway is at the center of the network, thus showing better performance. However, shorter MP2P and P2MP paths also imply that on average each node’s DODAG has a lower number of nodes, therefore there is a strong probability that many P2P data packets go through the gateway due to relatively longer P2P forwarding paths. The longer paths negatively impact P2P communications performance. Similarly, a gateway at the edge of the network results in longer MP2P and P2MP data forwarding paths, hence MP2P and P2MP communication PDR is negatively impacted. Longer paths result in relatively bigger DODAGs at most nodes, hence a lower number of P2P packets go through the gateway. This results in better P2P communication PDR.

The mean delay demonstrated by the protocols in different types of communication while the gateway was at the center of different random topologies is shown in Figure 7b. Generally, the trends present in Figure 7b and in Figure 6b are similar. However, there is one difference, i.e., due to shorter forwarding paths MP2P and P2MP communication experience lower delay.

The protocols’ performances comparison with regard to the number of times P2P packets are relayed to reach their destination and the total number of retransmissions is shown in Figure 7c,d, respectively. The trends shown in the figures are similar to the trends shown in Figure 6c,d, respectively.

#### 5.1.2. Results Using Grid Network Topology

**Figure 8:**
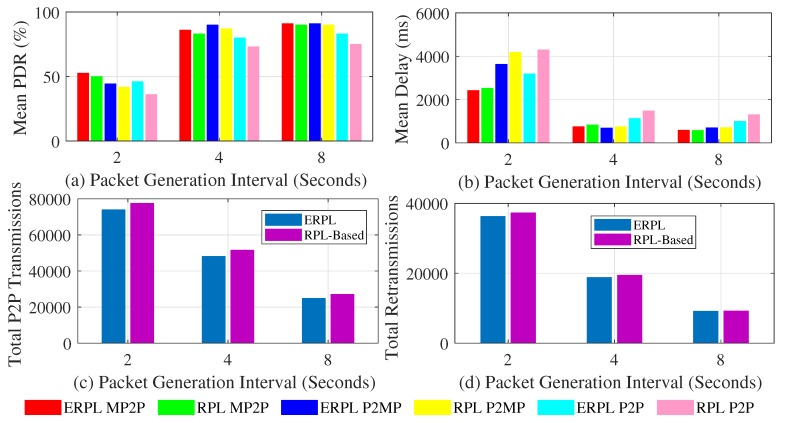
Protocols’ Performances with Grid Topology (Gateway at Network Edge).

Here we present the protocol’s performance based on a grid network topology. Figure 8 shows EPRL’s performance based on a grid topology when the gateway is placed at the center of the network. Figure 8a shows the mean PDR demonstrated by the protocols in MP2P, P2MP, and P2P communications. Compared to standard RPL, ERPL demonstrates higher PDR in all cases. The performance difference among the evaluated protocols increases as we decrease the data packet generation interval. The ERPL P2P forwarding algorithm tries to lowers the number of transmissions required to deliver a P2P data packet to its destination, hence the performance difference between the protocols increases as we decrease the data packet generation interval. ERPL’s enhanced P2P routing and forwarding algorithm also positively impacts MP2P and P2MP communication performance, which is similar to the random network topology results.

Figure 8b shows the mean per-packet end-to-end delay achieved by the protocols with MP2P, P2MP, and P2P communications. In all cases, ERPL demonstrates lower delay compared to standard RPL. The performance gap between the protocols increases as we decrease the packet generation interval. The reason for this is the same as discussed for the PDR results. With an increase in the packet generation interval both protocols demonstrate lower delay due to lower data traffic load in the network.

Figure 8c,d shows the two protocols’ performances with regard to the number of times P2P packets are relayed in the network and the total number of re-transmissions. The number of times P2P packets are relayed in the network using ERPL is lower compared to standard RPL. Moreover, ERPL demonstrates fewer re-transmissions. As ERPL relays P2P packets less, this results in a lower contention, hence ERPL demonstrates better PDR, delay, and total number of re-transmissions in the grid network topology as well.

**Figure 9:**
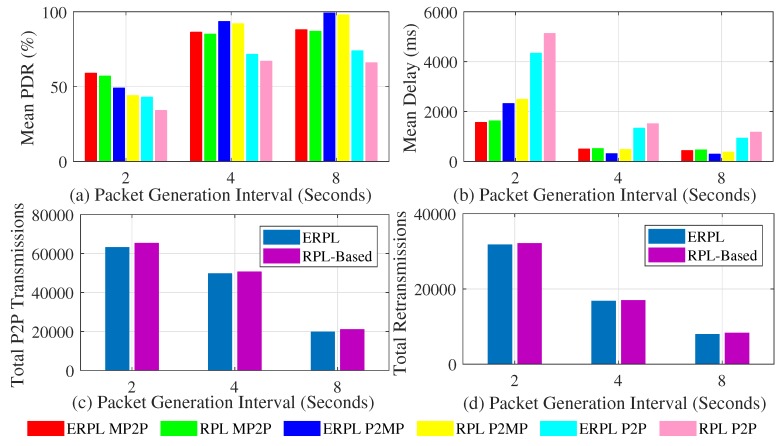
Protocols’ Performances with Grid Topology (Gateway at Network Center).

Figure 9 shows the performances of the two protocols based on the grid network topology when the gateway is placed at the network’s center. Figure 9a shows the two protocols’ PDR. The trends shown in Figure 9a are similar to the trends shown in Figure 8a. Comparison of Figure 8a and Figure 9a reveals that placing the gateway at the center of the network slightly improves MP2P and P2MP communications’ PDR, however the PDR corresponding to P2P communication is negatively impacted. This happens exactly due to the same reason that we discussed above for random network topologies.

Figure 9b shows the mean per-packet end-to-end delay achieved by the protocols with MP2P, P2MP, and P2P communications. The trends shown in Figure 9b are similar to the trends shown in Figure 8b. However, MP2P and P2MP communications experience lower delay compared to the delay experienced by the communications when the gateway was at the edge of the network. This is due to the fact that on average MP2P and P2MP forwarding paths are shorter when the gateway is at the center of the network.

Figure 9c,d show the protocols comparison with regard to the number of times P2P packets are relayed in the network and the total number of re-transmissions. The trends shown in Figure 9c,d are similar to the trends shown in Figure 8c,d respectively. Fewer total P2P transmissions associated with ERPL results in the protocol’s superior performance overall.

#### 5.1.3. Testbed Results

To validate our simulation-based results, we evaluated the protocols using the FlockLAB [24] test-bed. The experiments were performed using the FlockLAB’s TelosB motes. In the test-bed based experiments, each experiment duration and traffic generation model were the same as we used in our simulation based experiments. The current FlockLAB deployment consists of 30 nodes, however at the time of our experiments only twenty seven TelosB motes were operational. Twenty three nodes were deployed indoors across one floor in an office building, distributed in offices, hallways, and store rooms. Four nodes were deployed outside, placed on the roof of an adjacent building a few meters beneath the floor with the indoor nodes. In the test-bed based experiments, a node at the edge of the network was selected as the gateway.

**Figure 10:**
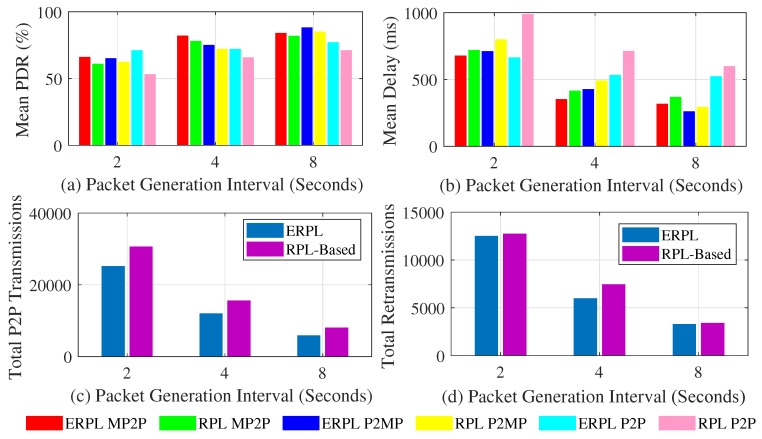
Protocols’ Performances in FlockLAB Testbed.

Figure 10a shows the PDR achieved by the evaluated protocols. The PDR results obtained through the test-bed validate our simulation based PDR results as the trends are similar, i.e., in all cases ERPL outperforms the standard RPL protocol. With a decrease in the packet generation interval the performance gap between the two protocols increases and both protocols show higher PDR with an increase in the packet generation interval.

Figure 10b shows the delay demonstrated by the two protocols. The delay results obtained through the test-bed again validate the results obtained through simulations as the trends are similar, i.e., in all cases ERPL outperforms the standard RPL protocol. Due to lower contention with an increase in the packet generation interval, both protocols demonstrate lower delay.

Figure 10c,d shows the performance of the two protocols with regard to the number of times P2P packets that are relayed in the network and the total number of re-transmissions. The test-bed results validate the simulation based results as again the trends are similar, i.e., ERPL demonstrates lower total P2P transmissions and re-transmission. Lower P2P transmissions associated with ERPL result in the protocol’s improved performance in all three communication types.

#### 5.1.4. Discussion

The proposed ERPL protocol reduces the number of times a P2P packet is relayed in a network in order to reach its destination. This enhancement is achieved without any additional control overhead, hence ERPL reduces the amount of contention in a network. This not only positively impacts the performance of P2P communication, but also positively impacts the performance of MP2P and P2MP communications. The protocol’s better P2P routing and forwarding algorithms result in lower energy consumption as the protocol also demonstrates fewer re-transmissions. Placing the gateway at the edge of the network results in longer MP2P and P2MP data forwarding paths, hence MP2P and P2MP performances are negatively impacted. However, at the same time it results in a relatively bigger DODAG at each node, therefore nodes can discover more peer nodes. Consequently, the number of times a P2P packet is relayed through the gateway is lowered. Similarly, placing the gateway at the center of a network results in shorter MP2P and P2MP data forwarding paths. It improves MP2P and P2MP communications, however due to a relatively shallow DODAGs most P2P packets travel through the gateway, hence P2P communication is negatively impacted.

### 5.2. ERPL’s P2MP Routing and Data Forwarding Performance Evaluation

Here, we present simulation results for our P2MP reduced overhead routing protocol. The idea here is to analyzes the actual impact of the Bloom Filter’s false positive rate on the protocol’s performance, therefore we did not use any radio propagation model in these set of simulations. We categorize our performance benchmarks as follows: reliability, source routing control overhead, and energy consumption. For reliability, we measure and report PDR. We compared our protocol’s performance against an ideal case, i.e., as we are not using any radio propagation model, hence we assume RPL delivers 100% packets to the destination node. We used a grid network topology with 50 nodes, and one node among those 50 nodes acts as the root node. In our simulations, the root transmits 10,000, 20,000, 30,000, 40,000, and 50,000 packets to different nodes in a network. Before transmitting a packet the root randomly selects a destination node and inserts the source routing information in the form of a BF into the packet header before transmitting the message. To obtain CI around the means, we repeated each set of simulations 30 times with different random seeds. Moreover, we used the following BF configurations: [m=128,k=4].

**Figure 11:**
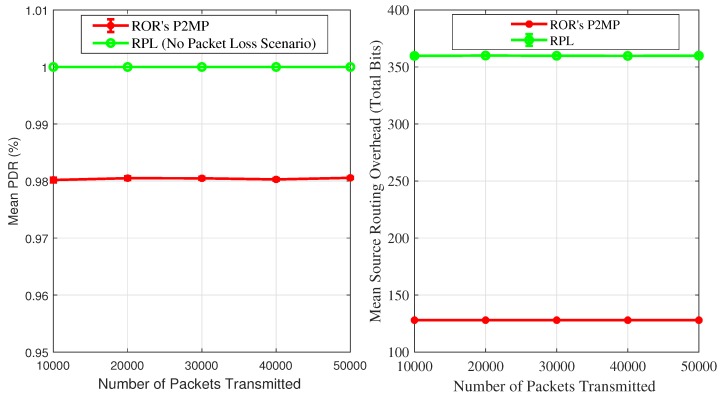
Performance Comparison.

Figure 11a shows the comparison of PDR achieved by our P2MP routing protocol in the ideal case. In different simulation scenarios, our P2MP routing protocol showed more than 98% PDR. The difference between the two protocols is minor, hence our PDR results demonstrate that the BF-based approach towards source routing can yield an acceptable level of performance. Use of a radio propagation model will reduce the PDR, however in that case the PDR for RPL will also be impacted. Figure 11b shows the mean per-packet source routing control overhead comparison. In order to obtain RPL’s source routing overhead we keep track of each data packet’s route length in our simulations and accumulate it for each transmitted packet. At the end of each simulation, we divide the accumulated route length value by the total transmissions to obtain the mean per-packet route length. We use IEEE 802.15.4 addresses in the source routing, therefore we multiplied the mean per-packet route length with 6 to obtain the mean number of overhead bytes per packet. For our P2MP reduced overhead routing, the control overhead is constant, i.e., 128 bits. On average, for each transmitted packet, our P2MP protocol demonstrated approximately 64% lower source routing control bits overhead than standard RPL. The mean per-packet overhead difference in terms of bits is 232 bits. Hence, using RPL’s source routing results in transmitting data packets that are bigger in size compared to our protocol. This can potentially negatively impact the PDR of RPL, and the difference among the protocol in terms of PDR will be further reduced. Moreover, it is a well-known fact that transmitting data packets consumes the highest amount of a node’s energy, therefore RPL’s energy consumption is likely to be substantially higher compared to our P2MP routing protocol.

Simulation results demonstrated that our P2MP routing protocol improves on RPL in the following aspects: fixed size address header, per-packet lower control overhead, and lower energy consumption. Moreover, the difference among the compared protocols in terms of PDR is very narrow. Furthermore, our simulation results also confirm that our P2MP reduced overhead routing protocol possesses the much required characteristics for a routing protocol for low-power and short-range wireless networks, such as lower energy consumption, lower overhead, and fixed size IP headers.

## 6. Conclusions

Standard RPL exhibits poor performance in P2P communication due to the required additional control overhead and inefficient P2P forwarding. Moreover, it uses source routing for P2MP communications, and possibly in P2P communications as well. The source routing results in variable size address headers, higher energy consumption, and possibly lower PDR due to higher control overhead. To address this, we presented ERPL, which is not only an enhanced routing and forwarding mechanism for P2P communication for RPL’s storing and non-storing MoPs, but it also presents a probabilistic forwarding mechanism based on BF to reduce source routing overhead for P2MP and P2P communications. ERPL P2P routing and forwarding algorithms reduce the number of transmissions required to delivery a P2P data packet to the destination node thus reducing delay and supporting delay sensitive Internet of Things and Cyber-Physical System applications over LLNs. Moreover, ERPL P2P routing and forwarding algorithms can coexist with existing RPL implementations. Our results demonstrate that ERPL P2P routing and forwarding algorithms not only outperform the standard RPL protocol in P2P communications, but they also outperform the protocol in MP2P and P2MP communications. Our results also demonstrate that ERPL’s probabilistic forwarding algorithm for P2MP communications substantially reduces the source routing overhead associated with RPL and hence better suits the requirements of short-range wireless LLNs.

## Figures and Tables

**Table 1:** Cooja Simulation Parameters.

Parameter	Value
MAC layer	IEEE 802.15.4 CSMA-CA
MAC layer ACKs	Enabled
Radio model	Unit disk with distance loss
Channel rate	250 kbps
MAC layer queue size	10 frames
Node transmission range	50 m
Node carrier sensing range	100 m
